# Ti_3_C_2_T_x_ (MXene) disrupts growth and development in *Daphnia magna* by suppressing related genes and inducing gut microbiome dysbiosis

**DOI:** 10.3389/fmicb.2026.1748570

**Published:** 2026-02-09

**Authors:** Qianqian Xiang, Yanping Wu, Yongfang Li, Shaoxiang Li, Xuexiu Chang

**Affiliations:** 1 Yunnan Collaborative Innovation Center for Plateau Lake Ecology and Environmental Health, College of Agronomy and Life Sciences, Kunming University, Kunming, China; 2 Great Lakes Institute for Environmental Research, University of Windsor, Windsor, ON, Canada

**Keywords:** *Daphnia magna*, gene expression, growth and development, gut microbiome, MXenes

## Abstract

The potential risks of Ti_3_C_2_T_x_ (MXene) nanomaterials to the ecological environment and human health have drawn increasing attention due to their widespread applications in the fields of biomedicine and environmental remediation. Although the aquatic ecotoxicity of Ti_3_C_2_T_x_ has been reported, little is known about how Ti_3_C_2_T_x_ disrupts the physiological processes that regulate growth and development in zooplankton. This study investigated the toxic effects and mechanisms of Ti_3_C_2_T_x_ exposure on the growth and development of *Daphnia magna* through gene expression and gut microbiome analyses. Results show that Ti_3_C_2_T_x_ exposure significantly reduced moulting frequency, body length, body width, and absolute growth rate in *D. magna*. Exposure to Ti_3_C_2_T_x_ led to a significant decrease in the expression of growth and development-related genes (*cyp18a1*, *ecra*, *usp, hr3*, and *cpa1*) in *D. magna*. Microbiome analysis revealed that exposure to Ti_3_C_2_T_x_ resulted in a decrease in Proteobacteria and an increase in Bacteroidota in the microbial community of *D. magna*. Meanwhile, Ti_3_C_2_T_x_ induced reduced abundances of *Pseudomonas* and *Aeromonas*, as well as increased abundances of *Bacillus* and *Phascolarctobacterium*. These microbial functions primarily contribute to energy acquisition and metabolism. This study indicated that Ti_3_C_2_T_x_ can inhibit the growth and development of *D. magna* by inhibiting the expression of growth and development-related genes and inducing intestinal microbial community dysbiosis. This study provides new insights into understanding the mechanisms of Ti_3_C_2_T_x_ toxicity on the growth and development of zooplankton in aquatic ecosystems.

## 1 Introduction

MXenes are an emerging class of two-dimensional transition metal carbon/nitride nanomaterials, first synthesized in 2011 (Naguib et al., 2011). The MXene family now encompasses over 30 distinct members, with Ti_3_C_2_T_x_ nanomaterials representing one of its most representative constituents (Cheng et al., 2022; Rong et al., 2024). Due to its unique physical and chemical properties, such as excellent metallic conductivity, hydrophilicity, dispersion stability, and flexibility, Ti_3_C_2_T_x_ has been extensively studied in fields including sensors (Kim et al., 2018), energy storage, medical therapy (Zhao et al., 2020), catalysis, and environmental remediation (Ganji et al., 2024; Liao et al., 2024; Ye et al., 2025). With large-scale production and immense applications anticipated in the foreseeable future, Ti_3_C_2_T_x_ materials will inevitably be released into the environment. It has raised public concerns regarding their potential risks to ecosystems and human health (Xie et al., 2020; Vasyukova et al., 2022).

Aquatic ecosystems are the ultimate destination for nanomaterials released into the environment. Consequently, understanding the ecotoxicity of Ti_3_C_2_T_x_ nanomaterials toward aquatic organisms is crucial for assessing their aquatic ecological safety and health impacts. The growth and development of aquatic organisms are important indicators for assessing environmental pollutants, and their abnormal changes will seriously affect the health of individual organisms and populations, and even threaten the structural and functional stability of aquatic ecosystems (Salerno et al., 2021). In recent years, the developmental toxicity of MXene toward aquatic organisms has been documented. Exposure to 100 and 200 μg/mL Ti_3_C_2_T_x_ for 4 days resulted in metallic titanium accumulation within zebrafish embryos, leading to increased mortality during embryonic development (Nasrallah et al., 2018). Similarly, exposure to 100 and 200 μg/mL Nb_2_CT_x_ and DL-Nb_4_C_3_T_x_ also induced mortality and malformations in zebrafish embryo development (Rasheed et al., 2024). Exposure to 5 and 10 μg/mL Ti_3_C_2_T_x_ for 7 days caused metabolic disruption in *Microcystis aeruginosa* (e.g., porphyrin and chlorophyll metabolism, glycerophospholipid metabolism), thereby inhibiting algal photosynthetic activity and ultimately suppressing algal growth (Xiang et al., 2025). These studies suggest MXene exposure poses potential threats to the growth and development of aquatic vertebrates and phytoplankton. However, little is known about how MXene disrupts the physiological processes that regulate growth and development in zooplankton.

*Daphnia magna* is one of the quintessential planktonic crustaceans within aquatic ecosystems (Ebert, 2022; Power et al., 2025). Owing to its diminutive size, prolific reproductive capacity, short life cycle, and acute sensitivity to water quality, *D. magna* has emerged as an ideal model organism for assessing nanomaterial contamination in aquatic environments (Roy and Roy, 2024; Queiroz and de Torresi, 2025). Exposure to Ti_3_C_2_T_x_ for 48 h induces substantial accumulation of metallic titanium within *D. magna*, resulting in acute mortality (Ye et al., 2025). Concurrently, 24-h exposure to Ti_3_C_2_T_x_ disrupted multiple metabolic pathways in *D. magna*, including phospholipids, pyrimidine, tryptophan, arginine, glycerol esters, and the pentose phosphate pathway (Xiang et al., 2024). Although the toxicity of Ti_3_C_2_T_x_ to *D. magna* has been explored, the effects of Ti_3_C_2_T_x_ on the growth and development of *D. magna* and their underlying mechanisms remain unclear. Some studies indicated that pollutants can influence *D. magna* growth and development by regulating gene expression associated with these processes (e.g., *cyp314*, *cyp18a1*, *ecra*, *usp*., *hr3*, *cut*, *cht*, and *cht3*) (Seyoum et al., 2020; Chen et al., 2021; Wei et al., 2022). Further investigations indicated pollutants can also disrupt the structure and function of *D. magna* microbial communities, thereby affecting growth and development (Akbar et al., 2020; Lovern and Van Hart, 2022). Evidently, examining gene expression patterns related to growth and development alongside microbial alterations offers novel perspectives for elucidating the mechanisms underpinning Ti_3_C_2_T_x_ toxicity toward *D. magna* growth and development.

The primary objective of this study is to investigate the toxic effects of Ti_3_C_2_T_x_ on the growth and development of *D. magna*, while integrating data on growth-related gene expression (*cyp314*, *cyp18a1*, *ecra*, *ecrb*, *usp*., *hr3*, *ftz-f1*, and *cpa1*) and gut microbiota (the community, diversity, and function of the microbiota) to elucidate the mechanisms underlying Ti_3_C_2_T_x_ toxicity. This study found that Ti_3_C_2_T_x_ exposure could interfere with the growth of *D. magna* and induce dysbiosis of its intestinal microbial community. These findings provide a reference for understanding the growth toxicity of Ti_3_C_2_T_x_ on planktonic crustaceans and its associated mechanisms.

## 2 Materials and methods

### 2.1 Experimental materials and organisms

Titanium carbide (Ti_3_C_2_T_x_) nanomaterials were purchased from Xianfeng Nano Technology Co., Ltd., Nanjing, China. The size and morphology of Ti_3_C_2_T_x_ were characterized using a transmission electron microscope (JIM-2100, Japan). The charge and hydrated size of Ti_3_C_2_T_x_ were determined using a Bru -Kehaven high-sensitivity Zeta potential and size analysis instruments (NanoBrook 90plus PALS, United States).

*Daphnia magna* was procured from the Guangdong Provincial Laboratory Animal Inspection Institute. Daphnia were continuously cultured for three generations in beakers containing tap water aerated for over 3 days within an artificial climate chamber (Shanghai Yiheng Scientific Instrument Co., Ltd.). Cultivation conditions were set as follows: light–dark cycle 16 h:8 h, temperature maintained at 21 ± 1 °C. Daphnia were fed twice daily with *Chlorella vulgaris*, at a concentration of 1 × 10^5^ cells/mL. These cultivation conditions comply with the protocol provided by the International Organization for Standardization (ISO 6341: 2012).

### 2.2 Experimental exposure protocol

This experiment established three Ti_3_C_2_T_x_ treatment groups: 0 mg/L (control group), 0.01 mg/L, and 1 mg/L. These concentrations were selected based on environmentally relevant titanium concentrations (Kaegi et al., 2008). To investigate the growth and development of *D. magna*, 1-day-old neonates (≤24 h) were selected because they are in the early developmental stage with high sensitivity to environmental pollutants, and their growth and development processes are relatively homogeneous, which can reduce the experimental variation caused by individual differences. 1-day-old neonates were exposed to three Ti_3_C_2_T_x_ treatment groups, with 5 individuals per 50 mL of exposure solution. The exposure period lasted for 7 days, during which Chlorella was fed daily at a concentration of 1 × 10^5^ cells/mL, and the exposure solution was completely renewed every 2 days. After the exposure period, *D. magna* was subjected to growth and development phenotypic analysis. Meanwhile, Daphnia samples were collected and stored in a − 80 °C ultra-low temperature refrigerator for subsequent analysis of the expression of growth and development-related genes. In addition, to obtain sufficient samples of the intestinal microbiota of *D. magna*, 14-day-old individuals with a stable gut microbiome were exposed following the same procedure described above. To avoid the interference of secondary changes in the gut microbiome caused by long-term growth inhibition, the daphnids were dissected under a stereomicroscope in a sterile environment after 1 day of exposure, and the intestines from every 20 individuals were pooled as one sample for intestinal microbiota analysis.

### 2.3 Observation of Ti_3_C_2_T_x_ accumulation in *Daphnia magna*

The accumulation of Ti_3_C_2_T_x_ within *D. magna* was observed and analyzed following the methods described in previous research (Xiang et al., 2024). Ti_3_C_2_T_x_, being a black substance, is readily observable and detectable within the transparent bodies of *D. magna*. Consequently, this study employed a standard optical microscope to observe Ti_3_C_2_T_x_ within *D. magna*. Briefly, Daphnia exposed for 7 days were absorbed onto concave glass slides and covered with coverslips. Samples were then placed under an Olympus optical microscope (Model BX53F2C) to observe Ti_3_C_2_T_x_ accumulation within the organisms. Distribution within the intestinal tract was documented using a CCD-D23 camera.

### 2.4 Analysis of the growth and developmental phenotypes of *Daphnia magna*

The growth and developmental phenotypes of *D. magna* were analyzed with reference to the methods reported in previous study (Qi et al., 2022). The primary biological indicators for growth and development in this study comprised moulting frequency, body length, body width, and absolute growth rate. Briefly, to determine the moulting frequency, *Daphnia* were observed and counted under a microscope every 24 h during the exposure period to record the number of molts. Following the conclusion of the 7-day exposure period, *Daphnia* were examined under an Olympus optical microscope (Model BX53F2C), with changes in body length and width documented and analyzed via CCD-D23 imaging. Concurrently, the absolute growth rate of *Daphnia* was analyzed based on body length data.

### 2.5 Determination of the expression of growth and development-related genes in *Daphnia magna*

The expression of growth and development-related genes in *D. magna* was analyzed with reference to the method reported in previous study (Chen et al., 2021). To evaluate growth and development progression, mRNA expression was measured for *cyp314*, *cyp18a1*, *ecra*, *ecrb*, *usp., hr3*, *ftz-f1*, and *cpa1* genes, which are critical regulators of *D. magna* growth and development. Briefly, total RNA was extracted from *D. magna* using the Trizol reagent kit according to the manufacturer’s protocol. Subsequently, the concentration and integrity of total RNA in each sample were analyzed using a nucleic acid and protein analyzer (NanoDrop2000) and agarose gel electrophoresis, respectively (Supplementary Figure S1; Supplementary Table S1). Finally, RNA was reverse transcribed into cDNA for quantitative real-time PCR detection. Primers for amplifying and detecting relevant genes in *D. magna* used in this study are provided in the Supplementary Table S2.

Real-time quantitative PCR (RT-qPCR) was analyzed using an ABI 7300 real-time quantitative PCR instrument (Applied Biosystems, United States). Briefly, First, the *Daphnia* cDNA obtained via reverse transcription was combined with primers upstream and downstream of the target gene and internal control gene. Subsequently, a 10 μL PCR reaction system was prepared using 2 × ChamQ SYBR Color qPCR Master Mix and sterile water following the SYBR Green I dye method. Reaction conditions were as follows: initial denaturation at 95 °C for 5 min, followed by 40 cycles comprising 95 °C for 5 s, 55 °C for 30 s, and 72 °C for 40 s. A melting curve analysis was subsequently performed to confirm reaction specificity. Finally, the *β-actin* gene from *D. magna* served as the reference internal control gene, and the relative mRNA expression levels of the target genes were calculated using the 2^-ΔΔCt^ method.

### 2.6 Gut microbial analysis of *Daphnia magna*

The analysis of the intestinal microbiota in *D. magna* was performed via high-throughput sequencing with reference to the method reported in previous study (Li et al., 2022). Briefly, DNA was first extracted from *Daphnia* intestinal samples using a DNA extraction kit (Omega Bio-tek, Norcross, GA, United States). Sample DNA integrity and concentration were assessed via 1% agarose gel electrophoresis and NanoDrop2000 (Thermo Scientific, United States), respectively (Supplementary Figure S1; Supplementary Table S3). Secondly, using the extracted DNA as template, PCR amplification of the 16S rRNA gene was performed with primers 341F (5’-CCTACGGGNGGCWGCAG-3′) and 785R (5’-GACTACHVGGGTATCTAATCC-3′). Subsequently, PCR products were recovered and purified via 2% agarose gel electrophoresis. Finally, recovered products were quantified using the Qubit 4.0 system (Thermo Fisher Scientific, United States) and sequenced on Shanghai Meiji Biotechnology Co., Ltd.’s Illumina PE300/PE250 platform.

Following sequencing completion, Operational Taxonomic Unit (OTU) clustering analysis was performed on quality-controlled, assembled sequences using 97% similarity. Concurrently, microbial community diversity indices were analyzed based on OTU data. Subsequently, OTU taxonomic annotation was performed using the Silva 16S rRNA gene database (v138) at a 70% confidence threshold, with community composition at various taxonomic levels quantified for each sample. Finally, functional prediction of microbial communities was analyzed using Tax4Fun. All microbial data analyses in this study were conducted on the Shanghai Meiji Bio Cloud Platform.^1^

### 2.7 Data analysis

The data from this study underwent normality testing via the Kolmogorov–Smirnov method. To distinguish significant differences between the control and treatment groups, a one-way analysis of variance (ANOVA) was performed using SPSS 26.0 software, followed by post-hoc multiple comparisons (Duncan’s test). A *p*-value < 0.05 indicates statistically significant differences between the control and treatment groups.

## 3 Results

### 3.1 Characterization of the physicochemical properties of Ti_3_C_2_T_x_ and its accumulation in *Daphnia magna*

Electron microscopy characterization revealed (Supplementary Figure S3) that the Ti_3_C_2_T_x_ exhibited the following typical features: the Ti_3_C_2_T_x_ material displayed distinct irregular flakes with a diameter of approximately 100 nm, showing no significant large-area agglomeration (Supplementary Figure S3A). The surface charge of Ti_3_C_2_T_x_ was negatively charged at approximately −18 mV, with a hydrated sheet diameter of approximately 1.8 μm. Furthermore, optical microscopy revealed (Supplementary Figure S3) that exposure to Ti_3_C_2_T_x_ can lead to their accumulation in *Daphnia*. No Ti_3_C_2_T_x_ was observed within the bodies of *D. magna* in the control group (Supplementary Figure S3B). However, Ti_3_C_2_T_x_ accumulation was observed in *D. magna* following exposure to both 0.01 and 1 mg/L concentrations, with the primary distribution occurring within intestinal tissues (Supplementary Figures S3C,D).

### 3.2 Phenotypic alterations in the growth and development of *Daphnia magna*

**Figure 1:**
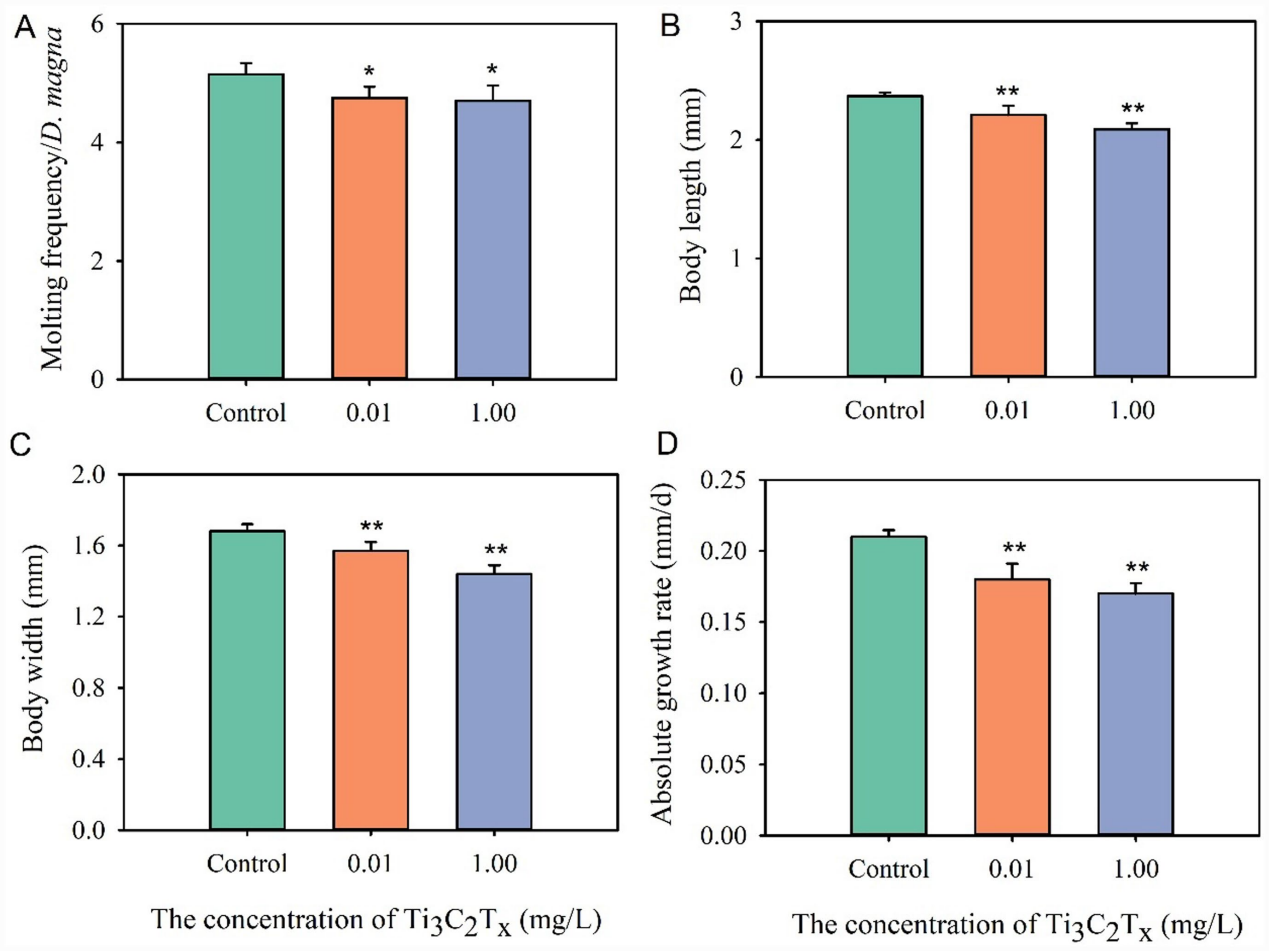
Effects of Ti_3_C_2_T_x_ on the growth and development phenotypes of *D. magna*. **(A)** Moulting frequency of *D. magna*. **(B)** Body length of *D. magna*. **(C)** Body width of *D. magna*. **(D)** Absolute growth rate of *D. magna*. *Indicates a statistically significant difference between the control group and treatment groups (*p* ≤ 0.05). **Indicates an extremely statistically significant difference between the control group and treatment groups (*p* ≤ 0.01).

Exposure to Ti_3_C_2_T_x_ can induce abnormal growth phenotypes in *D. magna* (Figure 1). Compared with the control group, exposure to 0.01 and 1 mg/L Ti_3_C_2_T_x_ significantly reduced the moulting frequency of *D. magna*. During the 7-day exposure period, the average moulting frequency per individual in the control group was 5.15 ± 0.19, while that in the 1 mg/L Ti_3_C_2_T_x_ group was 4.7 ± 0.26 (Figure 1A). Compared with the control group, exposure to both 0.01 and 1 mg/L Ti_3_C_2_T_x_ induced a significant reduction in the body length and width of *D. magna*. After 7 days of exposure, the average body length and width per individual in the control group were 2.37 ± 0.03 mm and 1.68 ± 0.04 mm, respectively, while those in the 1 mg/L Ti_3_C_2_T_x_ group were 2.09 ± 0.05 mm and 1.44 ± 0.05 mm (Figures 1B,C). Compared with the control group, exposure to 0.01 and 1 mg/L Ti_3_C_2_T_x_ both significantly reduced the absolute growth rate of *D. magna*. After 7 days of exposure, the average absolute growth rate in the control group was 0.21 ± 0.004 mm/d, while that in the 1 mg/L Ti_3_C_2_T_x_ group was 0.17 ± 0.007 mm/d (Figure 1D).

### 3.3 Gene expression related to *Daphnia magna* growth and development

**Figure 2:**
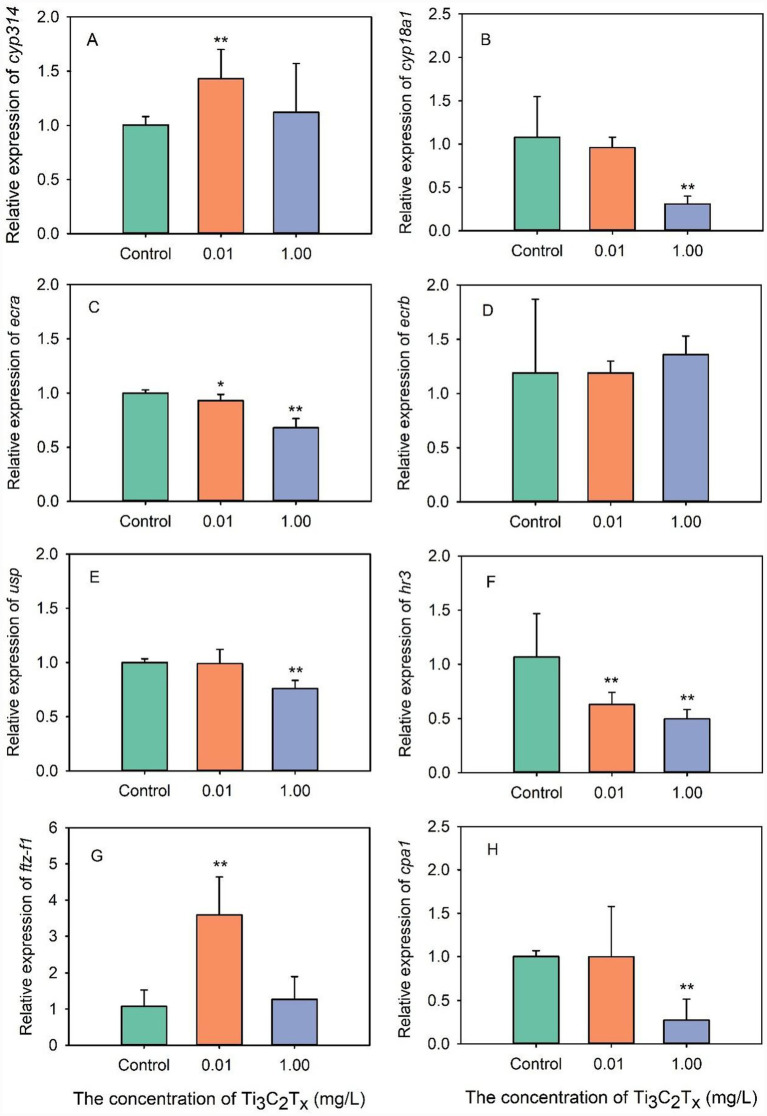
Effects of Ti_3_C_2_T_x_ on the expression of growth and development-related genes in *D. magna*. **(A)** The relative expression of *cyp314*. **(B)** The relative expression of *cyp18a1*. **(C)** The relative expression of *ecra*. **(D)** The relative expression of *ecrb*. **(E)** The relative expression of *usp*. **(F)** The relative expression of *hr3*. **(G)** The relative expression of *ftz-f1*. **(H)** The relative expression of *cpa1*.*Indicates a statistically significant difference between the control group and treatment groups (*p* ≤ 0.05). **Indicates an extremely statistically significant difference between the control group and treatment groups (*p* ≤ 0.01).

Exposure to Ti_3_C_2_T_x_ induced differential expression of genes related to growth and development in *D. magna* (Figure 2). Compared with the control group, exposure to 0.01 mg/L Ti_3_C_2_T_x_ significantly increased the expression of the growth and development genes *cyp314* and *ftz-f1* in *D. magna*, while significantly decreasing the expression of the genes *ecra* and *hr3* (Figures 2A,G,C,F). Notably, exposure to 1 mg/L Ti_3_C_2_T_x_ induced a significant reduction in the expression of the *D. magna* developmental genes *cyp18a1*, *ecra*, *usp., hr3*, and *cpa1*, while having no apparent effect on the expression of the genes *ecrb* and *ftz-f1* (Figures 2B–H). Exposure to both 0.01 and 1 mg/L Ti_3_C_2_T_x_ induced a significant reduction in expression of the *D. magna* developmental genes *ecra* and *hr3*.

### 3.4 Gut microbial community diversity

**Figure 3:**
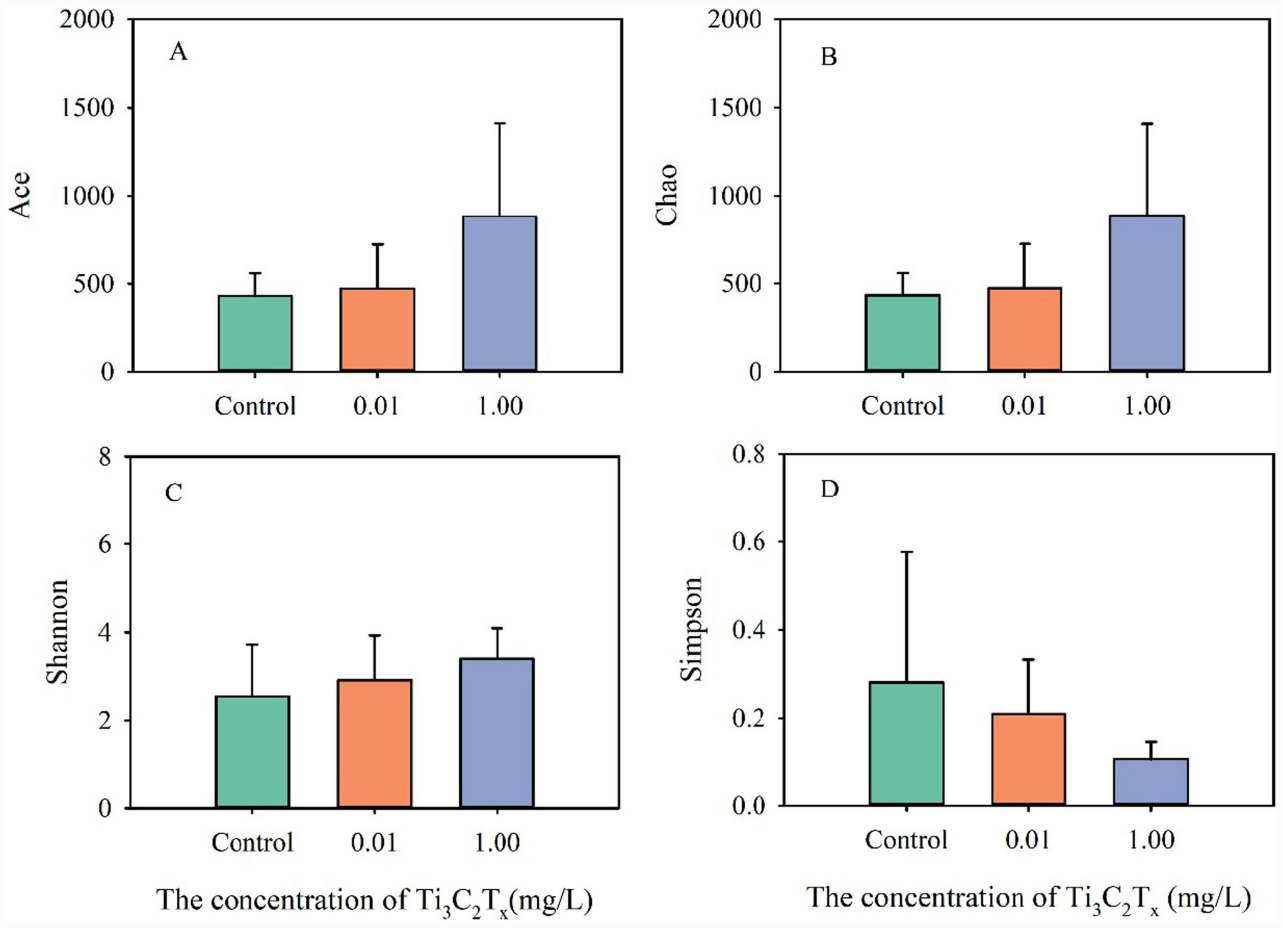
Effects of Ti_3_C_2_T_x_ on the *α*-diversity of the intestinal microbiota in *D. magna*. **(A)** Ace index, **(B)** Chao index, **(C)** Shannon index, **(D)** Simpson index.

Exposure to high concentrations of Ti_3_C_2_T_x_ showed a trend toward increased diversity and richness of the intestinal microbial community in *D. magna* (Figure 3). Compared to the control group, exposure to 0.01 mg/L Ti_3_C_2_T_x_ had no significant effect on Ace and Chao indices, whereas 1 mg/L exposure showed a trend toward increased Ace and Chao indices (Figures 3A,B). Similarly, exposure to 0.01 mg/L Ti_3_C_2_T_x_ showed no significant effect on the Shannon index, whereas 1 mg/L Ti_3_C_2_T_x_ exposure exhibited an increasing trend in the Shannon index (Figures 3C,D). Notably, exposure to 0.01 mg/L Ti_3_C_2_T_x_ resulted in a slight decrease in the Simpson index, whereas exposure to 1 mg/L Ti_3_C_2_T_x_ caused a significant reduction in the Simpson index.

### 3.5 Changes in gut microbial phyla and genera levels in *Daphnia magna*

**Figure 4:**
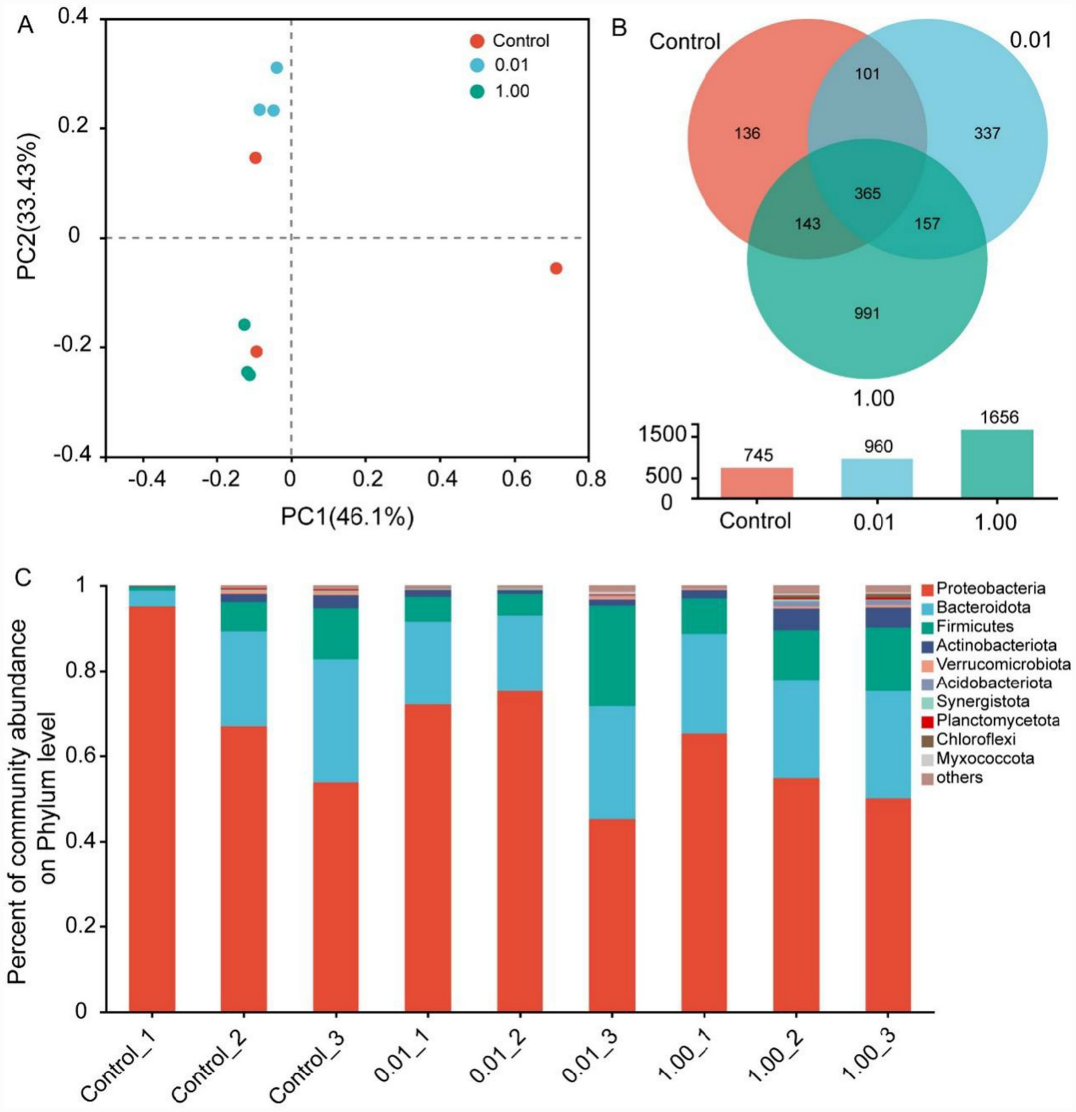
Ti_3_C_2_T_x_ exposure induces alterations in the intestinal microbiota of *D. magna*. **(A)** Principal coordinate analysis (PCoA) shows a clear separation of the intestinal microbiota between the control group and titanium carbide treatment groups. **(B)** Venn diagram displays the number of microbial species (i.e., OTUs) among different treatment groups. **(C)** 16S rRNA sequencing reveals the phylum-level composition profiles of the intestinal microbiota in the control group and titanium carbide treatment groups.

Exposure to Ti_3_C_2_T_x_ induced significant alterations in the phylum-level microbial communities of the intestinal tract in *D. magna* (Figure 4). Principal coordinate analysis (PCoA) results revealed (Figure 4A) that the first principal component accounted for 46.1% of variance, while the second component contributed 33.43%, indicating strong discriminative power between treatment groups. The Venn diagram results (Figure 4B) revealed 745, 960, and 1,656 OTUs in the control, 0.01 mg/L, and 1 mg/L Ti_3_C_2_T_x_ groups, respectively. Among these OTUs, 365 were shared among the different treatment groups (control group, 0.01 mg/L, and 1 mg/L), accounting for 16.37% of the total OTUs. In addition, the number of unique OTUs in the 0.01 mg/L Ti_3_C_2_T_x_ group was 337 (15.11%), while that in the 1 mg/L group was 991 (44.44%). Results at the microbial phylum level (Figure 4C) showed that in the control group, the most abundant phylum in the intestinal microbiota was Proteobacteria (71.94%), followed by Bacteroidota (18.35%), Firmicutes (6.53%), and Actinobacteriota (1.72%). In the 0.01 mg/L Ti_3_C_2_T_x_ group, Proteobacteria was still the dominant phylum (64.19%), followed by Bacteroidota (21.20%), Firmicutes (11.52%), and Actinobacteriota (1.31%). For the 1 mg/L Ti_3_C_2_T_x_ group, the phylum-level abundance ranking was Proteobacteria (56.71%), Bacteroidota (23.87%), Firmicutes (11.64%), and Actinobacteriota (3.90%).

Exposure to Ti_3_C_2_T_x_ induced significant changes in the intestinal microbiota community of *D. magna* at the genus level (Supplementary Figure S4). Compared with the control group, 0.01 mg/L Ti_3_C_2_T_x_ decreased the abundances of *Pseudomonas*, *Aeromonas*, and *Rhodobacteraceae* in the intestines of *D. magna*, while increasing the abundances of *Pedobacter* and *Bacillus*. In contrast, 1.00 mg/L Ti_3_C_2_T_x_ reduced the abundances of *Blastomonas*, *Pseudomonas*, *Aeromonas*, and *Chitinophagales*, and elevated the abundances of *Rhodobacteraceae*, *Bacillus*, *Acinetobacter*, and *Phascolarctobacterium* in the Daphnids’ intestines (Supplementary Figure S4).

### 3.6 Functional prediction of gut microbiota

**Figure 5:**
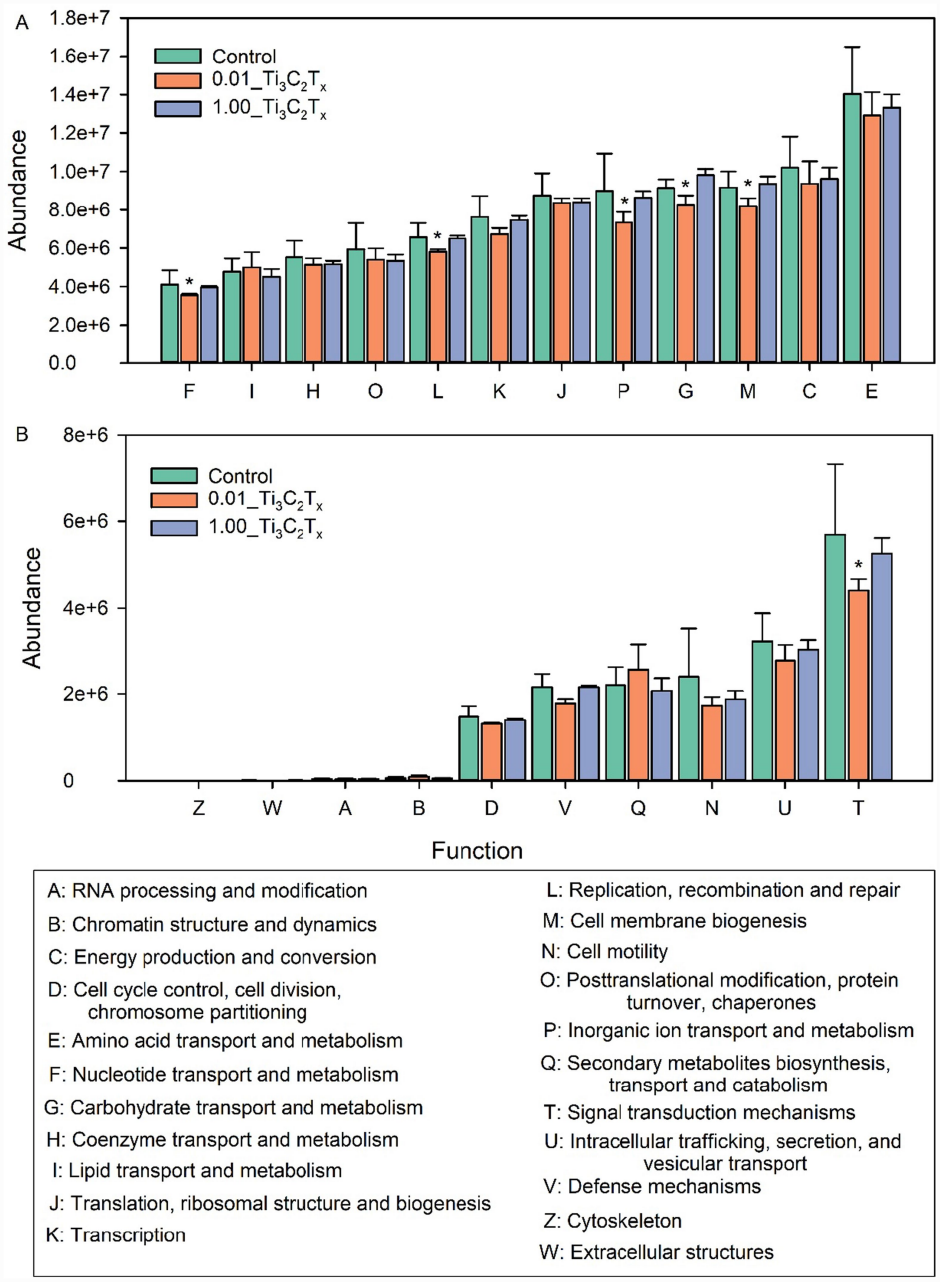
Functional prediction of intestinal microbiota involved in the growth and development of *D. magna* induced by Ti_3_C_2_T_x_. **(A)** Functional categories of intestinal microbiota directly involved in the growth and development of *D. magna* induced by Ti_3_C_2_T_x_. **(B)** Functional categories of intestinal microbiota indirectly involved in the growth and development of *D. magna* induced by Ti_3_C_2_T_x_. *Indicates a statistically significant difference between the control group and treatment groups (*p* ≤ 0.05).

Predictions of gut microbial functions indicated that Ti_3_C_2_T_x_ exposure induced significant differences in the abundance of gut microbial functional groups associated with the growth and development of *D. magna* (Figure 5). Among the functional categories directly related to the growth and development of *D. magna*, exposure to 0.01 and 1 mg/L Ti_3_C_2_T_x_ both decreased the abundances of amino acid transport and metabolism, energy production and conversion, and posttranslational modification, protein turnover, chaperones. Exposure to 0.01 mg/L Ti_3_C_2_T_x_ significantly reduced the abundances of inorganic ion transport and metabolism, replication, recombination and repair, cell membrane biogenesis, carbohydrate transport and metabolism, and nucleotide transport and metabolism (Figure 5A). For the functional categories indirectly associated with *D. magna* growth and development, exposure to 0.01 and 1 mg/L Ti_3_C_2_T_x_ both decreased the abundance of the cell motility functional category and increased that of the cytoskeleton functional category (Figure 5B).

## 4 Discussion

Nanomaterials exert a pronounced inhibitory effect on *D. magna* growth and development. As primary consumers in aquatic ecosystems, *Daphnia*’s developmental status directly influences food chain dynamics and material cycling equilibrium in freshwater ecosystems (Ebert, 2022; Liu et al., 2024). Consequently, *Daphnia*’s growth and development warrant significant attention. This study shown that exposure to Ti_3_C_2_T_x_ significantly reduced the moulting frequency, body length, and body width of *D. magna*, and even inhibits its growth rate. This indicates that exposure to Ti_3_C_2_T_x_ suppresses the growth and development of *D. magna*. Similarly, exposure to other metallic nanomaterials (e.g., titanium dioxide nanoparticles, iron oxide nanoparticles, silver nanoparticles, zinc oxide nanoparticles, copper nanoparticles, chromium nanoparticles) has been observed to reduce the moulting frequency, body length, and growth rate of *D. magna* (Lu et al., 2017; Ellis et al., 2020; Qi et al., 2022; Chorfi et al., 2024). Exposure to non-metallic nanomaterials (e.g., silica nanoparticles) also impairs *D. magna* growth (Kim et al., 2021). It is evident that the growth and development of *Daphnia* serve as important biological indicators for responding to nanomaterial pollution in aquatic environments. To the best of our knowledge, this study is the first to reveal the effects of Ti_3_C_2_T_x_ nanomaterials on the growth and development of *D. magna*.

Ti_3_C_2_T_x_ can inhibit the growth of *D. magna* by downregulating the expression of growth and development-related genes. As is well known, the growth and development of *D. magna* are closely associated with the expression of growth-related genes (Zhong et al., 2025). Previous study has shown that *cyp18a1*, *ecra*, *usp*., *hr3*, and *cpa1* were the major genes regulating the growth and development of *D. magna*, and their abnormal expression can directly affect the growth of *D. magna* (Chen et al., 2021). The present study found that Ti_3_C_2_T_x_ exposure significantly reduced the expression of *cyp18a1*, *ecra*, *usp*., *hr3*, and *cpa1* genes, accompanied by decreased growth. This suggests that Ti_3_C_2_T_x_ can reduce the growth of *D. magna* by inhibiting the expression of its growth-related genes. Similarly, perfluorooctane sulfonate (PFOS) exposure can reduce the growth of *D. magna* by downregulating the expression of *ecra*, *usp*., and *hr3* genes (Seyoum et al., 2020). Exposure to cadmium (Cd) can downregulate the *cyp18a1*, *ecra*, *usp*., *hr3*, and *cpa1* genes in *D. magna*, thereby inhibiting its growth (Wei et al., 2022). Exposure to polystyrene micro/nanoplastics also caused significant reduction in the expression of *D. magna* growth-related genes, resulting in diminished growth (Chen et al., 2024).

Dysbiosis of the intestinal microbiota is also one of the important factors through which Ti_3_C_2_T_x_ affects the growth and development of *D. magna*. The intestine is a key tissue and organ for digestion and absorption in organisms, and its microbiota plays a crucial role in regulating and maintaining the growth and development of the host (Li et al., 2023). Previous studies have shown that intestinal microbiota dysbiosis can cause abnormalities in the growth and development of organisms, mainly due to the microbiota’s involvement in the regulation of energy absorption and metabolism (Lovern and Van Hart, 2022; Qi et al., 2022). The present study found that exposure to Ti_3_C_2_T_x_ for 24 h induced intestinal microbiota dysbiosis, and was accompanied by decreased abundances of multiple microbial functions related to energy absorption and metabolism, such as amino acid transport and metabolism, energy production and conversion, inorganic ion transport and metabolism, and carbohydrate transport and metabolism. Similarly, our previous study has demonstrated that exposure to Ti_3_C_2_T_x_ can induce disturbances in the physiological energy absorption and metabolism of *D. magna* (Xiang et al., 2024). These results suggest that the early acute gut microbiome dysbiosis observed after 1-day exposure is likely an initial event leading to the 7-day growth inhibition phenotype in *D. magna*. The acute dysbiosis of intestinal microbiota induced by Ti_3_C_2_T_x_ may disrupt the energy supply process in the early stage of exposure. With the extension of exposure time, this initial disruption will accumulate and further affect the expression of growth-related genes, ultimately leading to the inhibition of growth and development. Other studies have demonstrated that carbon quantum dots (CQDs) can affect the growth and development of *D. magna* by disrupting the composition of the intestinal microbiota and reducing the abundances of microbial functions involved in energy absorption and metabolism (Ma et al., 2023). Zinc oxide nanoparticles can reduce the moulting frequency and body length of *D. magna* by downregulating its energy absorption and metabolic pathways (Qi et al., 2022).

The intestinal microbiota community structure of *D. magna* exhibits distinct sensitivities to different pollutants. The richness and diversity of the intestinal microbiota community are important biomarkers for organisms to adapt to the environment (Guo et al., 2025). The present study found that Ti_3_C_2_T_x_ exposure tended to increase the Ace, Chao, and Shannon indices, while decreasing the Simpson index. This indicates that Ti_3_C_2_T_x_ exposure slightly enhances the richness and diversity of the intestinal microbiota community in *D. magna*. In contrast, copper oxide nanoparticles exposure reduced the richness and diversity of the intestinal microbiota community in *D. magna* (Jin et al., 2024). Oxytetracycline exposure increased the richness and diversity of the intestinal microbiota community in *D. magna* (Lovern and Van Hart, 2022). Microfiber plastic exposure induced no significant changes in the richness and diversity of the microbiota community in *D. magna* (Lee et al., 2023). These studies suggest that the type of pollutant is one of the important factors affecting changes in the intestinal microbiota community structure of *D. magna*.

## 5 Conclusion

**Figure 6:**
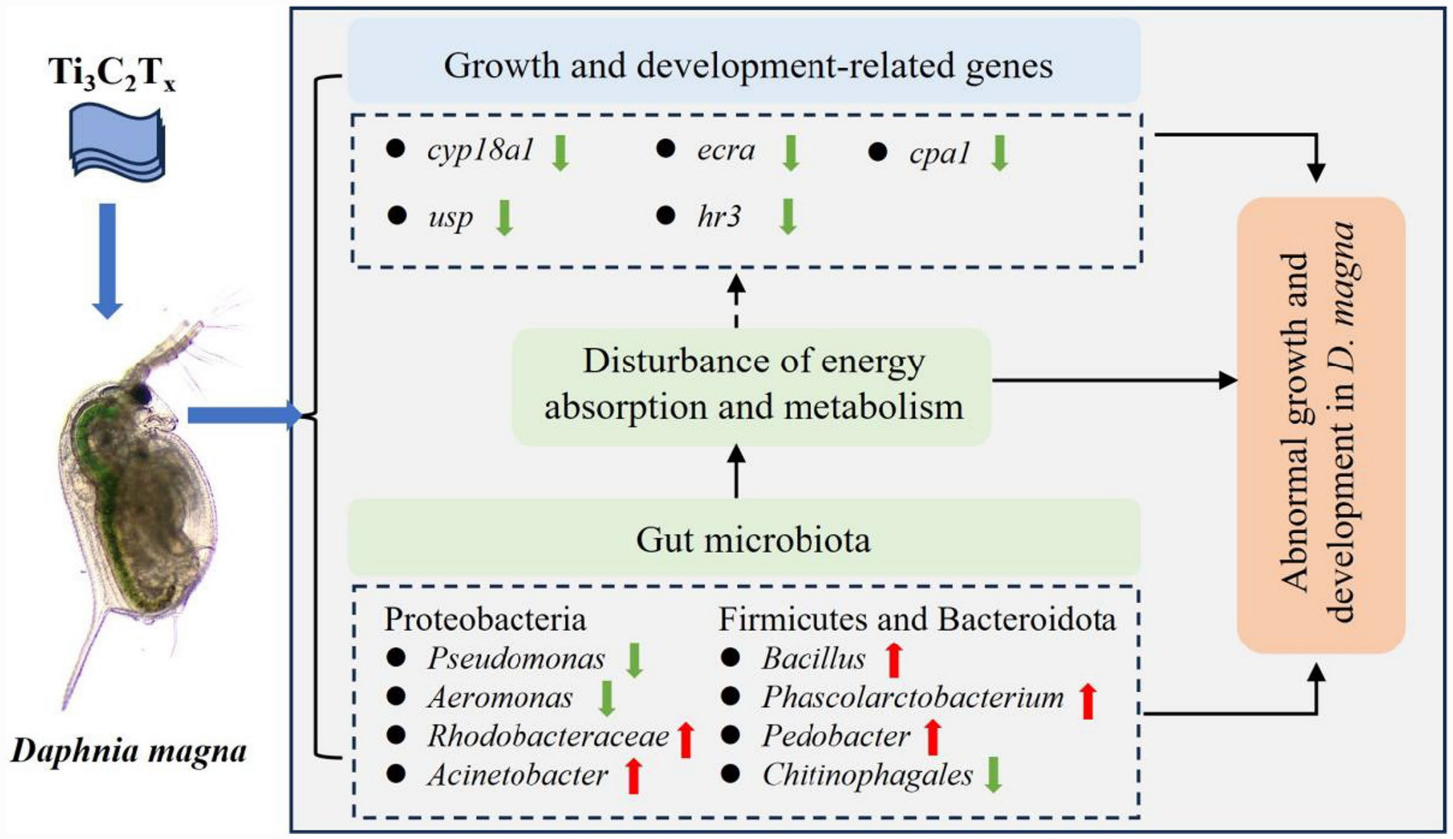
Schematic diagram of the potential mechanisms by which Ti_3_C_2_T_x_ induces abnormal growth and development in *D. magna*. Ti_3_C_2_T_x_ exposure induces two parallel but interactive toxicological processes in *D. magna*: (1) Directly downregulating the expression of growth-related genes, thereby inhibiting the growth and development process; (2) Inducing acute dysbiosis of the gut microbiome, which disrupts energy absorption and metabolism, and further indirectly affects the expression of growth-related genes. These two processes synergistically lead to the final growth inhibition phenotype.

This study is the first to reveal the toxic effects of Ti_3_C_2_T_x_ nanomaterials on the growth and development of *D. magna* through analyses at the genetic and microbial levels (Figure 6). Ti_3_C_2_T_x_ exposure can inhibit the growth and development of *D. magna* in a dose-dependent manner. 1 mg/L Ti_3_C_2_T_x_ exposure inhibited the growth of *D. magna* by downregulating the expression of genes *cyp18a1*, *ecra*, *usp., cpa1* and *hr3*. Meanwhile, 1 mg/L Ti_3_C_2_T_x_ exposure also reduced the abundances of *Pseudomonas* and *Aeromonas*, and increased the abundances of *Rhodobacteraceae*, *Bacillus*, and *Acinetobacter* in the *Daphnids’* intestines, disrupting energy absorption and metabolism, thereby affecting the growth and development of *D. magna* (Figure 6). The intestinal microbiota community structure of *D. magna* shows marked differences in sensitivity to different pollutants. Although this study investigated the growth and developmental toxicity of Ti_3_C_2_T_x_ to *D. magna* across multiple toxic endpoints, it has certain limitations. Specifically, detailed information on the surface termination groups (-O, -OH, -F) of Ti_3_C_2_T_x_ is lacking, which may introduce certain uncertainties in interpreting its observed toxic effects on *D. magna*. Future studies should prioritize using Ti_3_C_2_T_x_ with well-characterized surface termination groups to further elucidate the structure–activity relationship between its surface properties and toxicological effects. This study provides new insights into the toxic mechanisms of Ti_3_C_2_T_x_ nanomaterials on the growth and development of typical planktonic crustaceans.

## Data Availability

The datasets presented in this study can be found in online repositories. The raw microbiological sequencing reads were deposited into NCBI Sequence Read Archive (SRA) database (Accession Number: SRP665408).

## Footnotes

1
https://cloud.majorbio.com
