# Supplementary material for: Ti3C2Tx (MXene) disrupts growth and development in Daphnia magna by suppressing related genes and inducing gut microbiome dysbiosis
Source: Front Microbiol. 2026 Feb 9;17:1748570. doi: 10.3389/fmicb.2026.1748570 (PMC12926406; doi:10.3389/fmicb.2026.1748570)
Supplement: Supplementary file 1 [file Supplementary_file_1.docx]

**Supporting Information**

**Ti_3_C_2_T_x_ (MXene) disrupts growth and development in *Daphnia magna* by suppressing related genes and inducing gut microbiome dysbiosis**

Qianqian Xiang^a*^, Yanping Wu^a^, Yongfang Li^a^, Shaoxiang Li^a^, Xuexiu Chang^a, b*^

^a^Yunnan Collaborative Innovation Center for Plateau Lake Ecology and Environmental Health, College of Agronomy and Life Sciences, Kunming University, Kunming 650214, China

^b^Great Lakes Institute for Environmental Research, University of Windsor, Windsor, ON N9B 3P4, Canada

^*^ Corresponding Author: Xuexiu Chang, E-mail: [xchang@uwindsor.ca](mailto:xchang@uwindsor.ca); Qianqian Xiang, E-mail: [xiangqqian@163.com](mailto:xiangqqian@163.com).


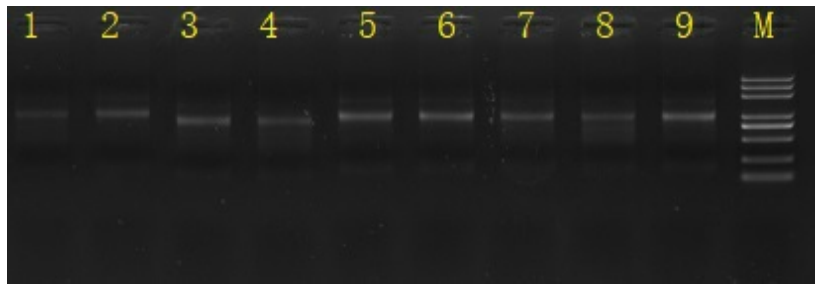


**Figure S1 Total RNA integrity of Daphnia tissue samples.** 1-3 represent the control group; 4-6 represent the 0.01 mg/L Ti_3_C_2_T_x_ group; 7-9 represent the 1 mg/L Ti_3_C_2_T_x_. group.


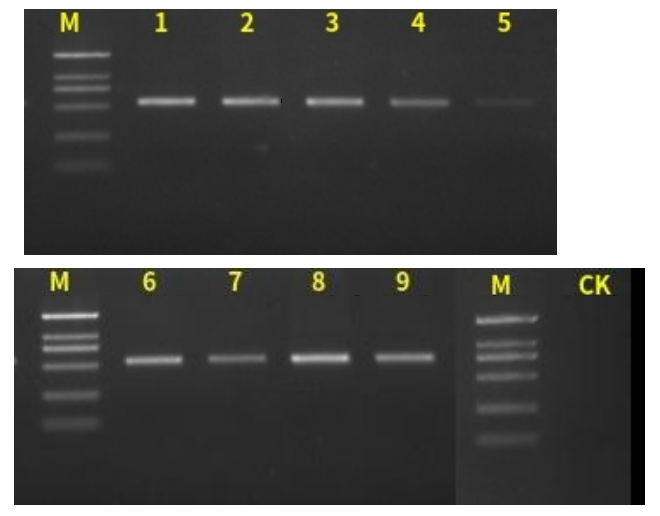


**Figure S2 Total DNA integrity of Daphnia intestinal microbial samples.** 1-3 represent the control group; 4-6 represent the 0.01 mg/L Ti_3_C_2_T_x_ group; 7–9 represent the 1 mg/L Ti_3_C_2_T_x_ group.


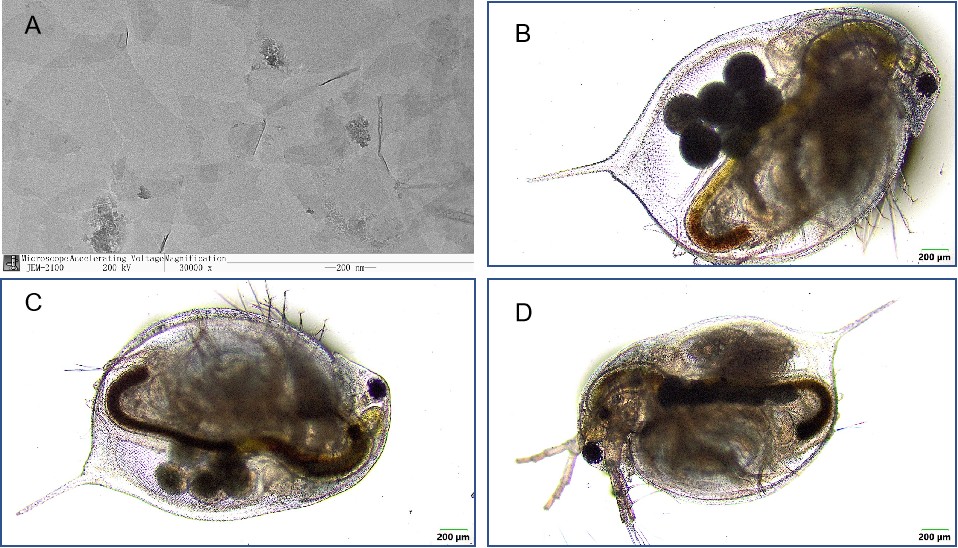


**Figure S3 Morphological characterization of Ti_3_C_2_T_x_ and its accumulation in *Daphnia magna*.** (A) Transmission electron micrograph of Ti_3_C_2_T_x_ material. (B) Control group. (C) 0.01 mg/L Ti_3_C_2_T_x_ group. (D) 1 mg/L Ti_3_C_2_T_x_ group.


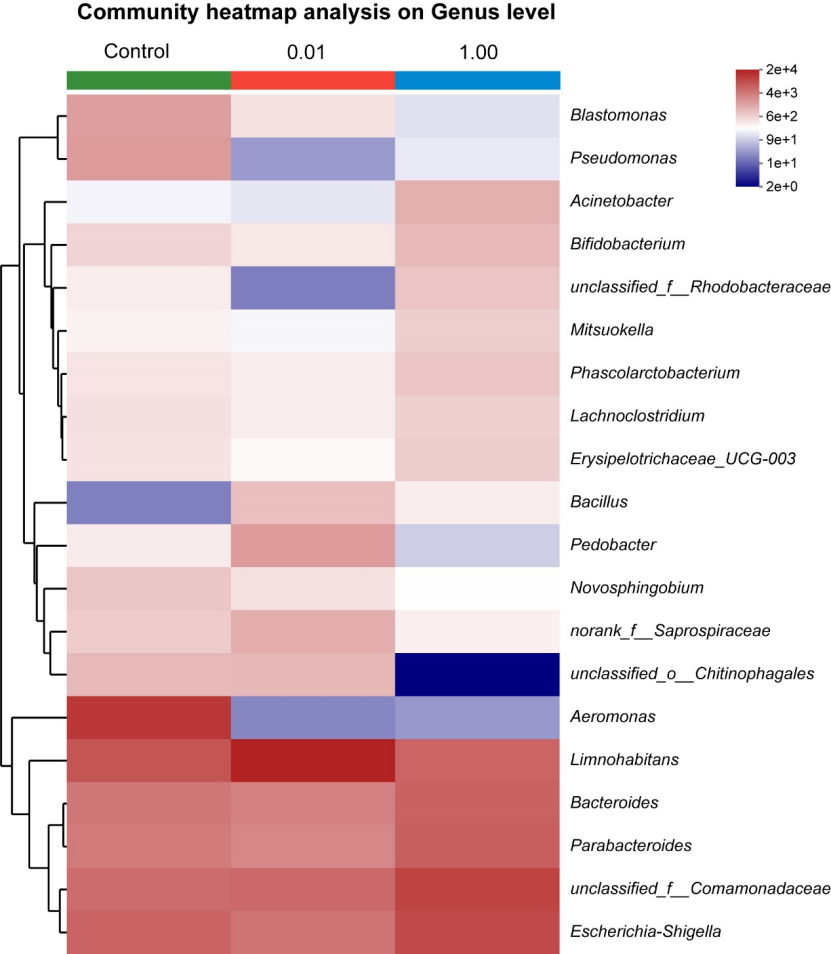


**Figure S4 Effects of Ti_3_C_2_T_x_ on gut microbial communities at the genus level in *Daphnia magna*.**

**Table S1 Total RNA concentration in *Daphnia magna* tissue samples**

| Sample name | Concentration (ng/μL) | OD260/280 |
| --- | --- | --- |
| Control | 46.20±8.32 | 2.01±0.026 |
| 0.01 | 55.77±1.79 | 2.02±0.015 |
| 1.00 | 51.70±4.27 | 2.06±0.03 |

**Table S2 Primer utilized for the growth and development of *Daphnia magna* for qRT-PCR.**

| Gene | Primer sequence （5’-3’） | Refs |
| --- | --- | --- |
| *cyp314* | F: ACTATGTATGGACTTCCCTGGTG | (Jo et al., 2018) |
|  | R: TTATCGCGGGTGTCAACG |  |
| *cyp18a1* | F: TACCCGATCGTCGGTTACCT | (Giraudo et al., 2017) |
|  | R: GAGCGCCGTCAGCTCTTC |  |
| *ecra* | F: CAGTTCGTCCATGTCGATGAG | (Blewett et al., 2017) |
|  | R: CGAAGGCGGTAAGGTAGAATG |  |
| *ecrb* | F: CACCACAACCAACTGCATTTAC | (Kato et al., 2007) |
|  | R: CCATTAATGTCAAGATCCCACA |  |
| *usp* | F: TAGGCCACTCGGGTTACTTAAA | (Kato et al., 2007) |
|  | R: GAGTGGGTGGTTAGGTGGATAA |  |
| *hr3* | F: AAGGTCGAGGATGAAGTGCG | (Giraudo et al., 2017) |
|  | R: AAAGACGCTACTATCGGGCG |  |
| *ftz-f1* | F: TCTTACCGGACATTCACGCC | (Giraudo et al., 2017) |
|  | R: ACAGCCGTTGAGATGCTTGA |  |
| *cpa1* | F: CCGACATTCACGTCAGT | (Blewett et al., 2017) |
|  | R: CCTCGTAGGTGTGATAGTT |  |
| *β-actin* | F: GCCCTCTTCCAGCCCTCATTCT | (Nkoom et al., 2022) |
|  | R: TGGGGCAAGGGCGGTGATTT |  |

S3 Total DNA concentration of intestinal microbial samples from *Daphnia magna.*

| Sample Name | Concentration (ng/μL) | OD260/280 |
| --- | --- | --- |
| Control | 3.17±1.45 | 2.15±0.61 |
| 0.01 | 2.83±1.56 | 2.83±0.05 |
| 1.00 | 4.10±0.44 | 2.87±0.06 |

**References**

Blewett, T.A., Delompré, P.L.M., He, Y.H., Folkerts, E.J., Flynn, S.L., Alessi, D.S., Goss, G.G., 2017. Sublethal and reproductive effects of acute and chronic exposure to flowback and produced water from hydraulic fracturing on the water flea *Daphnia magna*. Environ. Sci. Technol. 51, 3032-3039. <https://doi:10.1021/acs.est.6b05179>.

Giraudo, M., Douville, M., Cottin, G., Houde, M., 2017. Transcriptomic, cellular and life-history responses of *Daphnia magna* chronically exposed to benzotriazoles: endocrine-disrupting potential and molting effects. PLoS One. 12, e0171763. <https://doi:10.1371/journal.pone.0171763>.

Jo, M., Lee, S., Yoon, S., Kim, W.K., 2018. Developmental and reproductive effects of tamoxifen on *Daphnia magna*. Environ. Monit. Assess. 190, 677. <https://doi:10.1007/s10661-018-7002-y>.

Kato, Y., Kobayashi, K., Oda, S., Tatarazako, N., Watanabe, H., Iguchi, T., 2007. Cloning and characterization of the ecdysone receptor and ultraspiracle protein from the water flea *Daphnia magna*. J. Endocrinol. 193, 183-194. <https://doi:10.1677/joe-06-0228>.

Nkoom, M., Lu, G.H., Liu, J.C., 2022. Chronic toxicity of diclofenac, carbamazepine and their mixture to *Daphnia magna*: a comparative two-generational study. Environ. Sci. Pollut. R. 29, 58963-58979. <https://doi:10.1007/s11356-022-19463-w>.
